# Optimal UAV Formation Tracking Control with Dynamic Leading Velocity and Network-Induced Delays

**DOI:** 10.3390/e24020305

**Published:** 2022-02-21

**Authors:** Zhuwei Wang, Mengjiao Xu, Lihan Liu, Chao Fang, Yang Sun, Huamin Chen

**Affiliations:** 1 Faculty of Information Technology, Beijing University of Technology, Beijing 100124, China; wangzhuwei@bjut.edu.cn (Z.W.); xumengjiao@emails.bjut.edu.cn (M.X.); fangchao@bjut.edu.cn (C.F.); sunyang@bjut.edu.cn (Y.S.); chenhuamin@bjut.edu.cn (H.C.); 2 School of Information, Beijing Wuzi University, Beijing 101149, China; 3 Purple Mountain Laboratory: Networking, Communications and Security, Nanjing 210096, China

**Keywords:** formation tracking, high dynamic, leader–follower control, network-induced delays

## Abstract

With the rapid development of UAV technology, the research of optimal UAV formation tracking has been extensively studied. However, the high maneuverability and dynamic network topology of UAVs make formation tracking control much more difficult. In this paper, considering the highly dynamic features of uncertain time-varying leader velocity and network-induced delays, the optimal formation control algorithms for both near-equilibrium and general dynamic control cases are developed. First, the discrete-time error dynamics of UAV leader–follower models are analyzed. Next, a linear quadratic optimization problem is formulated with the objective of minimizing the errors between the desired and actual states consisting of velocity and position information of the follower. The optimal formation tracking problem of near-equilibrium cases is addressed by using a backward recursion method, and then the results are further extended to the general dynamic case where the leader moves at an uncertain time-varying velocity. Additionally, angle deviations are investigated, and it is proved that the similar state dynamics to the general case can be derived and the principle of control strategy design can be maintained. By using actual real-world data, numerical experiments verify the effectiveness of the proposed optimal UAV formation-tracking algorithm in both near-equilibrium and dynamic control cases in the presence of network-induced delays.

## 1. Introduction

UAVs have been extensively studied and have attracted more and more attention due to their high maneuverability and versatility [1,2,3]. For example, UAVs can be used as an air communication platform to provide or improve communication services for ground equipment and can also be used as air relays to address issues such as information transmission, monitoring, and control. Currently, UAVs with communication capabilities have been widely used in many scenarios, such as communication assistance, intelligent transportation, disaster rescue, and low-altitude monitoring [4,5,6,7,8].

However, a single UAV is limited by both battery energy and action scale constraints, thus causing challenges in mission implementation capabilities. Compared with a single UAV, the coordination of multiple UAVs brings advantages such as high efficiency, robustness, and flexibility. UAV swarms have a wide range of potential application, especially in highly reconfigurable and distributed intelligence autonomous systems [9,10,11]. Leader–follower formation control, as the most classic formation method to ensure successful mission execution, has the simplest formation control structure and requires the fewest communication connections between UAVs [12,13,14]. Although extensive studies have been performed on leader–follower formation control, it still faces unique challenges relating to the requirements of real-time application [15,16]. First, how to effectively address the influence of network-induced delays on the multi-dimensional formation control of UAVs is a new challenge. Network-induced delays degrade the performance of UAV systems and bring potential drawbacks to the systems’ stability [17,18]. Most existing works focus on stability analysis considering network-induced delays instead of effective control strategy design. For example, in some works, Lyapunov–Krasovskii functionals and Young’s inequalities are normally used in UAV control design to achieve stability for the whole formation. Making matters worse, the high mobility and dynamic nature of UAV application scenarios make the formation control of UAVs much more complicated and difficult. Considering the dynamic leader velocity, the measurement information may not be consistent with the current state of the leader, thus increasing the difficulty of designing a real-time control strategy.

Unfortunately, there is little research that focuses on the scenario which considers both time-varying leader velocity and network-induced delays, which are the fundamental dynamic natures introduced by the high mobility of UAVs [19]. In this paper, the optimal UAV formation tracking control design is comprehensively investigated for the leader–follower model in the discrete-time domain with dynamic leader velocity and network-induced delays. First, a linear discrete-time model of UAVs’ error dynamics is formulated. Subsequently, the optimal formation tracking control problem of near-equilibrium cases is addressed with stable leader velocity. Finally, the results are further extended to the general time-varying velocity case, and the effect of angle deviation is also analyzed. The main contributions are summarized as follows.

- Based on the analysis of UAVs’ error dynamics, considering the high dynamic characteristics including both uncertain time-varying leader velocity and network-induced delays, the discrete-time UAV system model is presented. Then, the formation tracking optimization problem is formulated as a linear quadratic cost function.
- To alleviate the influence of dynamic features, a two-step optimal formation tracking control algorithm is proposed in near-equilibrium control cases. That is, the optimal control strategy determined by the current states of the UAVs and previous control signals can be obtained during the online step, while the corresponding control gain is derived during the offline step by using backward recursion.
- Additionally, it is found that the proposed optimal control algorithm can be extended to the general dynamic case when the leader has dynamic time-varying velocity. Finally, the angle deviations are investigated, and it is proved that the similar state dynamics as the general case can be derived, thus the principle of the proposed control strategy for the general dynamic case can be maintained.
- Numerical experiment results based on real UAV flight data demonstrate that the proposed optimal UAV formation-tracking algorithm is applicable to general dynamic control cases in the presence of network-induced delays. In addition, compared with existing algorithms, faster convergence speed and better system stability are achieved.

The rest of this paper is organized as follows. In Section 2, some related works are reviewed. In Section 3, the system model and problem formulation are presented. Afterward, the formation tracking control algorithm is proposed in Section 4. Section 5 is the simulation results and discussion, and the conclusion is presented in Section 6.

## 2. Related Works

Network-induced delays significantly degrade the system performance and control stability of UAV formation tracking [20]. At present, significant efforts have been made to compensate for the influence of network-induced delays on UAV control. Lin et al. [21] study the consensus problem of a continuous-time multi-agent system with delay and jointly connected topology, and then a linear delay-based protocol is proposed and the sufficient condition for average consensus is achieved. When the input delay is considered, Zhu et al. [22] propose an event-based leader–follower consensus algorithm, and the necessary and sufficient conditions are presented. Considering both time delays and switching topology, the necessary and sufficient conditions for finite-field consensus of networks are derived by Li [23], subject to limited computation, memory, and communication capabilities. Then, the authors further extend the results to the multi-agent system in [24]. Currently, Chen et al. [25] use the Lyapunov–Krasovskii functionals and Young’s inequalities in the design process for the leader–follower consensus problem to eliminate the effects of network-induced delays.

In addition, due to the challenge of high mobility UAV applications, the dynamic formation control problem with system uncertainty has been considerable interest [26,27,28,29]. Olfati-Saber et al. [30] propose a distributed algorithm for flocking control that allows all groups of UAVs to ultimately achieve the same velocity, and the result is further expanded in [31] to show that the velocity and position of the center of the UAV swarm can exponentially converge on the virtual leader. In order to alleviate the velocity information requirements of the leader, Ghommam et al. [32] propose an adaptive feedback control algorithm restricted to parametric uncertainties of LOS-based leader–follower formations. Necessary and sufficient conditions for UAV swarm systems to achieve time-varying formations are developed in [33], but the time-delay is ignored. Currently, Yazdani et al. [34] design a continuous adaptive controller to solve the flocking problem of a multi-agent system with a dynamic virtual leader, which is also affected by both time-varying uncertainty and external interference. Considering both constant and time-varying velocities of the leader, a distributed coordinated tracking control scheme with network-induced delay and external interference is proposed in [35].

The comparison with relevant existing works can be summarized as in Table 1, in which “Yes” means that the related item is studied in the existing work, while “No” means that it is not. Different from these existing works, both dynamic leading velocity and network-induced delays are investigated in this paper. Additionally, an optimal control strategy for UAV formation tracking control under these conditions is derived.

## 3. System Model and Problem Formulation

**Figure 1:**
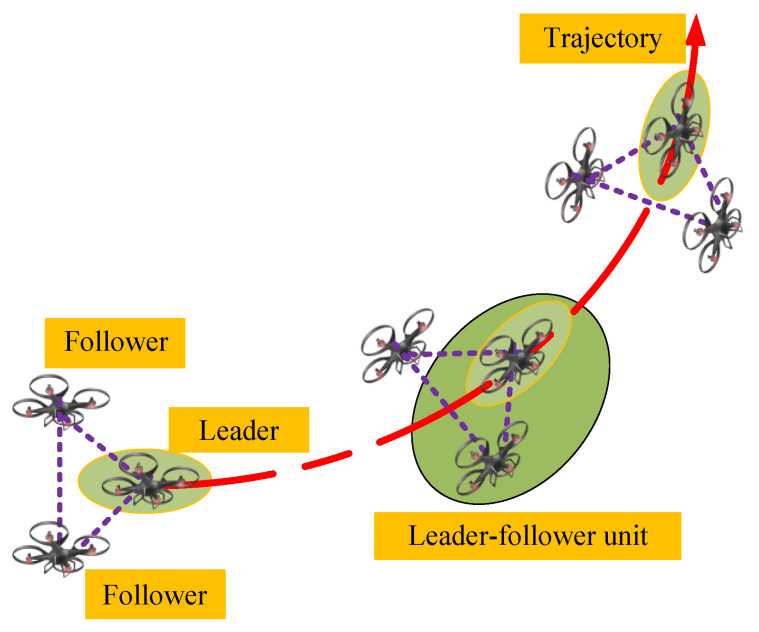
UAV formation tracking model.

**Figure 2:**
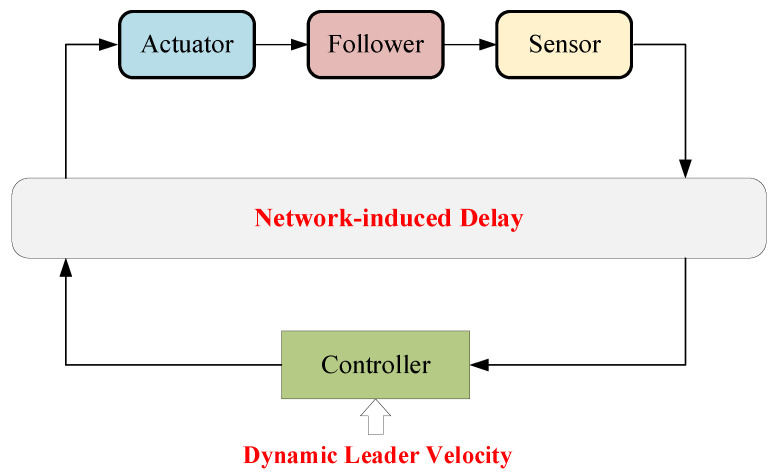
Dynamic features.

Figure 1 shows a typical 3D-based formation tracking control model. The leader UAV is controlled by a control station with dynamic velocity, and follower UAVs keep the desired formation while tracking with the leader. The state information of the leader is measured through sensors and transmitted by wireless sensor agent networks. Then, the control signal is generated depending on the measured information [1,3]. In this scenario, each UAV takes the nearest UAV as the tracking target and follows it, so the whole formation can be divided into several basic leader–follower units. Nevertheless, due to the high mobility of UAVs, the leader always has dynamic velocity and acceleration. Thereby, two fundamental challenges arise. One is how to guarantee that the follower keeps up with the leader smoothly under uncertain time-varying leader velocity. The other is how to reduce the effect of network-induced delays. The features of the dynamic leader velocity and network-induced delays are depicted in Figure 2, which significantly affects the design of the controller.

The dynamics of a follower are given by

(1) $$\begin{array}{l} \dot{l}(t)=p(t),\\ \dot{p}(t)=u\left(t-\Delta T(t)\right), \end{array}$$

where $l\left(t\right)={\left[l_{x}\left(t\right),\;l_{y}\left(t\right),\;l_{z}\left(t\right)\right]}^{T}$ and $p\left(t\right)={\left[p_{x}\left(t\right),\;p_{y}\left(t\right),\;p_{z}\left(t\right)\right]}^{T}$ are the position and velocity of the follower, respectively; $u\left(t\right)={\left[u_{x}\left(t\right),\;u_{y}\left(t\right),\;u_{z}\left(t\right)\right]}^{T}$ represents the follower’s acceleration, namely the formation tracking control strategy; ΔT(t) is the network-induced delay, which mainly includes the leader-to-controller delay, controller-to-follower delay, and signal processing delay.

The dynamics of desired states can be described by two integrators [31]

(2) $$\begin{array}{l} {\dot{l}}_{r}\left(t\right)=p_{r}\left(t\right),\\ {\dot{p}}_{r}\left(t\right)=g_{r}\left(l_{r}\left(t\right),p_{r}\left(t\right)\right), \end{array}$$

where $p_{r}\left(t\right)={\left[p_{rx}\left(t\right),\;p_{ry}\left(t\right),\;p_{rz}\left(t\right)\right]}^{T}$ and $l_{r}\left(t\right)={\left[l_{rx}\left(t\right),\;l_{ry}\left(t\right),\;l_{rz}\left(t\right)\right]}^{T}$ are the desired time-varying velocity and position information of leader, respectively, which are wholly determined by the leader’s state; $g_{r}\left(l_{r}\left(t\right),p_{r}\left(t\right)\right)$ represents the leader’s acceleration.

**Figure 3:**
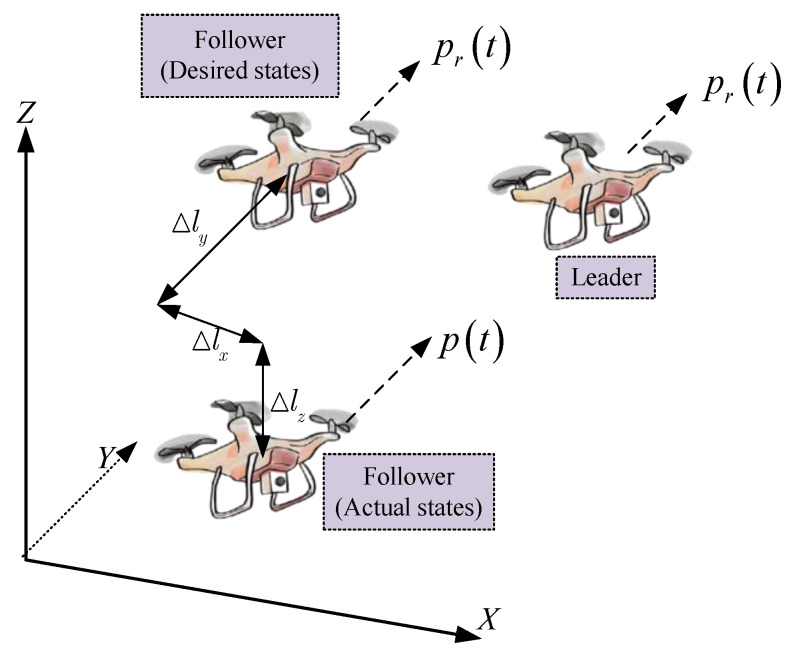
Leader–follower model for UAV formation.

The leader–follower model for UAV formation is shown in Figure 3. The objective for the follower is to track the reference trajectory generated by the leader and keep a desired time-varying formation. Define the velocity and position errors as

(3) $$\begin{array}{l} \Delta p\left(t\right)=p\left(t\right)-p_{r}\left(t\right),\\ \Delta l\left(t\right)=l\left(t\right)-l_{r}\left(t\right). \end{array}$$

Then, the error dynamics of follower formation tracking can be derived as

(4) $$\begin{array}{l} \Delta \dot{l}\left(t\right)=\Delta p\left(t\right),\\ \Delta \dot{p}\left(t\right)=u\left(t-\Delta T\left(t\right)\right)-g_{r}\left(l_{r}\left(t\right),p_{r}\left(t\right)\right). \end{array}$$

Define the state vector as

(5) $$s\left(t\right)={\left[\Delta l_{x}\left(t\right),\;\Delta p_{x}\left(t\right),\;\Delta l_{y}\left(t\right),\;\Delta p_{y}\left(t\right),\;\Delta l_{z}\left(t\right),\;\Delta p_{z}\left(t\right)\right]}^{T}.$$

Based on the error dynamics, the formation tracking model can be expressed as

(6) $$\dot{s}\left(t\right)=As\left(t\right)+B\left[u\left(t-\Delta T\left(t\right)\right)-g_{r}\left(l_{r}\left(t\right),p_{r}\left(t\right)\right)\right],$$

where

(7) $$\begin{array}{l} A=\left[\begin{array}{ccc} \bar{A} & 0_{2\times 2} & 0_{2\times 2}\\ 0_{2\times 2} & \bar{A} & 0_{2\times 2}\\ 0_{2\times 2} & 0_{2\times 2} & \bar{A} \end{array}\right],\\ B=\left[\begin{array}{ccc} \bar{B} & 0_{2\times 1} & 0_{2\times 1}\\ 0_{2\times 1} & \bar{B} & 0_{2\times 1}\\ 0_{2\times 1} & 0_{2\times 1} & \bar{B} \end{array}\right], \end{array}$$

$0_{i\times j}$ denotes the i×j zero matrix, and the block matrices are given by

(8) $$\bar{A}=\left[\begin{array}{cc} 0 & 1\\ 0 & 0 \end{array}\right],\;\bar{B}=\left[\begin{array}{c} 0\\ 1 \end{array}\right].$$

**Figure 4:**
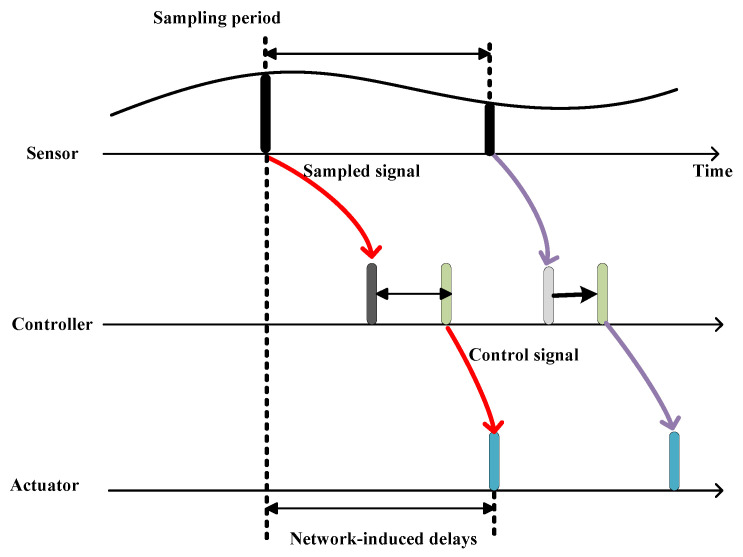
Timing diagram for UAV formation tracking through a wireless communication network.

Figure 4 shows the timing diagram for the UAV formation tracking control. The continuous-domain UAV states are first sampled, and then transmitted to the controller. Based on the received state information, the generated control signal is finally transmitted to the actuator to improve the formation tracking of the follower. In the control procession, it can be seen that network-induced delays from the sensor to the controller and from the controller to the actuator are caused, which are typically assumed to be stochastic.

Then, the corresponding discrete-time dynamics in the j-th sampling interval [jT, (j+1)T) can be derived as (see derivation details in Appendix A)

(9) $$s_{j+1}=Es_{j}+D_{j}^{1}u_{j}+D_{j}^{2}u_{j-1}+\delta_{j},$$

where

(10) $$\begin{array}{l} s_{j}=s\left(jT\right),\;E=e^{AT},\\ D_{j}^{1}=\int_{0}^{T-\Delta T_{j}}e^{At}dtB,\;D_{j}^{2}=\int_{T-\Delta T_{j}}^{T}e^{At}dtB,\\ \delta_{j}=-\int_{jT}^{\left(j+1\right)T}e^{A\left[\left(j+1\right)T-t\right]}g_{r}\left(l_{r}\left(t\right),p_{r}\left(t\right)\right)dtB, \end{array}$$

$u_{j}$ is the control signal generated based on system state $s_{j}$, and $\Delta T_{j}$ is the network-induced delay in j-th sampling interval, which is typically assumed to be stochastic.

Note that due to the inherent high mobility of the leader, the network-induced delay $\Delta T_{j}$ causes the time-varying feature of $D_{j}^{1}$ and $D_{j}^{2}$. On the other hand, the dynamic leader’s acceleration introduces the uncertain item $\delta_{j}$. The objective of the formation tracking control is to minimize the errors between desired and actual states of the follower and to maintain smooth control of the follower. Therefore, using the typical quadratic cost function, the optimization formation tracking problem in the dynamic leader–follower system can be formulated as

(11) $$\begin{array}{l} \underset{\left\{u_{j}\right\}}{\min}\;E\left[s_{N}^{T}Qs_{N}+\sum_{j=0}^{N-1}\left(s_{j}^{T}Qs_{j}+u_{j}^{T}Ru_{j}\right)\right]\\ s.t.\;s_{j+1}=Es_{j}+D_{j}^{1}u_{j}+D_{j}^{2}u_{j-1}+\delta_{j}, \end{array}$$

where N is the finite time horizon, Q and R are determined system parameters, E is the expectation operator due to the stochastic nature of the leader’s velocity and network-induced delays, $s_{j}^{T}Qs_{j}$ represents the tracking errors, and $u_{j}^{T}Ru_{j}$ denotes the effect of follower acceleration on preventing harsh control reactions.

## 4. Formation Tracking Control Algorithm

In this section, in order to solve the formation tracking optimization problem in (11), a near-equilibrium case in which the leader moves along a straight line at a near-constant velocity is considered at first. The optimal control strategy is derived by a two-step algorithm through backward iteration. Then, the results are extended to the general dynamic case in which the leader moves with an uncertain time-varying velocity. Lastly, the angle deviation is analyzed.

### 4.1. Near-Equilibrium Control Strategy Design

In most of the cases, the formation tracking system remains steady so that the leader flies smoothly at a near-constant velocity and the follower attempts to maintain its stable formation throughout tracking the reference trajectory. That is to say, the leader velocity keeps a near-constant value of $p_{r}\left(t\right)=p^{*}+\Delta$, where Δ→0. In this near-equilibrium case, the acceleration is $g_{r}\left(l_{r}\left(t\right),p_{r}\left(t\right)\right)\approx 0$ and the network-induced delay is $\Delta T_{j}\approx \Delta T$. That is, the uncertain item $\delta_{j}$ is approximately equal to zero.

Then, the optimization problem (11) can be simplified as

(12) $$\begin{array}{l} \underset{\left\{u_{j}\right\}}{\min}\;s_{N}^{T}Qs_{N}+\sum_{j=0}^{N-1}\left(s_{j}^{T}Qs_{j}+u_{j}^{T}Ru_{j}\right)\\ s.t.\;s_{j+1}=Es_{j}+D^{1}u_{j}+D^{2}u_{j-1}, \end{array}$$

where $D^{1}$ and $D^{2}$ become the determined parameters.

Let

(13) $$x_{j}=\left[\begin{array}{c} s_{j}\\ u_{j-1} \end{array}\right].$$

Then, the discrete-time dynamics can be rewritten as

(14) $$x_{j+1}=Fx_{j}+Hu_{j},$$

where

(15) $$F=\left[\begin{array}{cc} E & D^{2}\\ 0_{3\times 6} & 0_{3\times 3} \end{array}\right],\;H=\left[\begin{array}{c} D^{1}\\ I_{3\times 3} \end{array}\right],$$

and $I_{i\times i}$ denotes the i×i identity matrix.

Subsequently, the optimization problem (12) is equivalent to

(16) $$\begin{array}{l} \underset{\left\{u_{j}\right\}}{\min}\;x_{N}^{T}\bar{Q}x_{N}+\sum_{j=0}^{N-1}\left(x_{j}^{T}\bar{Q}x_{j}+u_{j}^{T}Ru_{j}\right)\\ s.t.\;x_{j+1}=Fx_{j}+Hu_{j}, \end{array}$$

where

(17) $$\bar{Q}=\left[\begin{array}{cc} Q & 0_{6\times 3}\\ 0_{3\times 6} & 0_{3\times 3} \end{array}\right].\;$$

**Theorem** **1.** *The optimal control strategy for the near-equilibrium formation tracking problem (12) is given by*

(18) $$u_{j}^{*}=-L_{j}x_{j},\;j=0,\;1,\ldots ,\;N-1,$$

*where* $L_{j}$*is iteratively calculated by*

(19) $$\begin{array}{l} L_{j}={\left[H^{T}S_{j+1}H+R\right]}^{-1}H^{T}S_{j+1}F,\\ S_{j}=F^{T}S_{j+1}F+\bar{Q}-L_{j}^{T}H^{T}S_{j+1}F,\\ S_{N}=\bar{Q}. \end{array}$$

The proof can be achieved similarly to the derivation process of optimal control strategy in [6].

### 4.2. General Dynamic Control Strategy Design

In general, the leader flies with a time-varying velocity with a highly dynamic state. Then, the uncertain time-varying term $\delta_{j}$, typically assumed to be a stochastic variable with zero mean value and variance matrix $\sigma_{\delta }$, should be considered together with the time-varying terms $D_{j}^{1}$ and $D_{j}^{2}$ in the optimal formation tracking control design.

Based on the definition $x_{j}={\left[s_{j}^{T},\;u_{j-1}^{T}\right]}^{T}$ in (13), the dynamic formation tracking control problem (11) can be rewritten as

(20) $$\begin{array}{l} \underset{\left\{u_{j}\right\}}{\min}\;E\left[x_{N}^{T}\bar{Q}x_{N}+\sum_{j=0}^{N-1}\left(x_{j}^{T}\bar{Q}x_{j}+u_{j}^{T}Ru_{j}\right)\right]\\ s.t.\;x_{j+1}={\tilde{F}}_{j}x_{j}+{\tilde{H}}_{j}u_{j}+{\left[\delta_{j}^{T},0_{3\times 1}\right]}^{T}, \end{array}$$

where ${\tilde{F}}_{j}$, ${\tilde{H}}_{j}$, and $\delta_{j}$ are time-varying items that

(21) $${\tilde{F}}_{j}=\left[\begin{array}{cc} E & D_{j}^{2}\\ 0_{3\times 6} & 0_{3\times 3} \end{array}\right],\;{\tilde{H}}_{j}=\left[\begin{array}{c} D_{j}^{1}\\ I_{3\times 3} \end{array}\right].$$

Define the residual cost function as

(22) $$V_{j}=\underset{\left\{u_{i}\right\}}{\min}E\left[x_{N}^{T}\bar{Q}x_{N}+\sum_{i=j}^{N-1}\left(x_{i}^{T}\bar{Q}x_{i}+u_{i}^{T}Ru_{i}\right)\right].$$

**Theorem** **2.** *The optimal control design for the general dynamic formation tracking problem (20) can be similarly derived as*

(23) $${\tilde{u}}_{j}^{*}=-{\tilde{L}}_{j}x_{j},\;j=0,\;1,\ldots ,\;N-1,\;$$

*where* ${\tilde{L}}_{j}$ *can be iteratively calculated by*

(24) $$\begin{array}{l} {\tilde{L}}_{j}=E{\left[{\tilde{H}}_{j}^{T}{\tilde{S}}_{j+1}{\tilde{H}}_{j}+R\right]}^{-1}E\left[{\tilde{H}}_{j}^{T}{\tilde{S}}_{j+1}{\tilde{F}}_{j}\right],\\ {\tilde{S}}_{j}=E\left[{\tilde{F}}_{j}^{T}{\tilde{S}}_{j+1}{\tilde{F}}_{j}\right]+\bar{Q}-{\tilde{L}}_{j}^{T}E\left[{\tilde{H}}_{j}^{T}{\tilde{S}}_{j+1}{\tilde{F}}_{j}\right],\\ {\tilde{S}}_{N}=\bar{Q}, \end{array}$$

*and the corresponding residual cost function is given by*

(25) $${\tilde{V}}_{j}=E\left[x_{j}^{T}{\tilde{S}}_{j}x_{j}\right]+\sum_{i=j+1}^{N}tr\left({\tilde{S}}_{i}^{1,1}\sigma_{\delta }\right).$$

*where* ${\tilde{S}}_{i}^{1,1}$ *denotes the* (1,1)−th *block of* ${\tilde{S}}_{i}$ *with the same size as* $\delta_{i}$ *, and* tr(⋅) *is the trace of the matrix.*

**Proof** **of** **Theorem** **2.** When j=N−1: ${\tilde{V}}_{N-1}$ can be deduced by

(26) $$\begin{array}{ll} {\tilde{V}}_{N-1} & =\underset{{\tilde{u}}_{N-1}}{\min}E\left[{\left[\begin{array}{c} x_{N-1}\\ u_{N-1} \end{array}\right]}^{T}\tilde{ℜ}\left[\begin{array}{c} x_{N-1}\\ u_{N-1} \end{array}\right]\right]+E\left[\delta_{N-1}^{T}{\tilde{S}}_{N}^{1,1}\delta_{N-1}^{}\right]\\ & =E\underset{{\tilde{u}}_{N-1}}{\min}\left[{\left[\begin{array}{c} x_{N-1}\\ u_{N-1} \end{array}\right]}^{T}\tilde{ℜ}\left[\begin{array}{c} x_{N-1}\\ u_{N-1} \end{array}\right]\right]+tr\left({\tilde{S}}_{N}^{1,1}\sigma_{\delta }\right), \end{array}$$

where

(27) $$\tilde{ℜ}=\left[\begin{array}{cc} b_{1,1} & b_{1,2}^{T}\\ b_{2,1} & b_{2,2} \end{array}\right],$$

that

(28) $$\begin{array}{l} b_{1,1}=E\left[{\tilde{F}}_{N-1}^{T}{\tilde{S}}_{N}{\tilde{F}}_{N-1}\right]+\bar{Q},\\ b_{2,2}=E\left[{\tilde{H}}_{N-1}^{T}{\tilde{S}}_{N}{\tilde{H}}_{N-1}\right]+R,\\ b_{2,1}=E\left[{\tilde{H}}_{N-1}^{T}{\tilde{S}}_{N}{\tilde{F}}_{N-1}\right]. \end{array}$$

Accordingly, the optimal control strategy can be derived as

(29) $${\tilde{u}}_{N-1}^{*}=-{\tilde{L}}_{N-1}x_{N-1},$$

where

(30) $${\tilde{L}}_{N-1}=E{\left[{\tilde{H}}_{N-1}^{T}{\tilde{S}}_{N}{\tilde{H}}_{N-1}+R\right]}^{-1}E\left[{\tilde{H}}_{N-1}^{T}{\tilde{S}}_{N}{\tilde{F}}_{N-1}\right],$$

and the corresponding residual cost function has the quadratic form

(31) $${\tilde{V}}_{N-1}=E\left[x_{N-1}^{T}{\tilde{S}}_{N-1}x_{N-1}\right]+tr\left({\tilde{S}}_{N}^{1,1}\sigma_{\delta }\right).$$

When j=N−2, …, 1, 0: assuming ${\tilde{V}}_{i}$, i≥j+1 also has the quadratic form

(32) $${\tilde{V}}_{j}=E\left[x_{j}^{T}{\tilde{S}}_{j}x_{j}\right]+\sum_{i=j+1}^{N}tr\left({\tilde{S}}_{i}^{1,1}\sigma_{\delta }\right).$$

Similar to the derivation from (16) to (24) in [6], the optimal formation tracking control strategy for general dynamic cases can be given by

(33) $${\tilde{u}}_{j}^{*}=-{\tilde{L}}_{j}x_{j},$$

where ${\tilde{L}}_{j}$ can be deduced iteratively on the basis of ${\tilde{S}}_{j+1}$ as in (24), and

(34) $${\tilde{V}}_{j}=E\left[x_{j}^{T}{\tilde{S}}_{j}x_{j}\right]+\sum_{i=j+1}^{N-1}tr\left({\tilde{S}}_{i}^{1,1}\sigma_{\delta }\right).$$

□

Thus, when the uncertain item $\delta_{k}$ is considered, an extra cost item $\sum_{i=j+1}^{N}tr\left({\tilde{S}}_{i}^{1,1}\sigma_{\delta }\right)$ is introduced in the residual cost function. Although time-varying items ${\tilde{F}}_{j}$, ${\tilde{H}}_{j}$ and $\delta_{j}$ introduced by the highly dynamic features of the leader exist, the same form of control strategy can be obtained in two steps in a backward recursion manner, which is summarized as Algorithm 1.
**Algorithm 1:** Formation Tracking Control Design.1   **Step 1: off-line**2   Initialize ${\tilde{S}}_{N}=\bar{Q}.$
3   **for** j=N−1:−1:0
**do**4   Calculate ${\tilde{L}}_{j}$ by using5   ${\tilde{L}}_{j}=E{\left[{\tilde{H}}_{j}^{T}{\tilde{S}}_{j+1}{\tilde{H}}_{j}+R\right]}^{-1}E\left[{\tilde{H}}_{j}^{T}{\tilde{S}}_{j+1}{\tilde{F}}_{j}\right].$
6   Calculate ${\tilde{S}}_{j}$ by using7   ${\tilde{S}}_{j}=E\left[{\tilde{F}}_{j}^{T}{\tilde{S}}_{j+1}{\tilde{F}}_{j}\right]+\bar{Q}-{\tilde{L}}_{j}^{T}E\left[{\tilde{H}}_{j}^{T}{\tilde{S}}_{j+1}{\tilde{F}}_{j}\right].$
8   **end**9   **Step 2: On-line**10  Initialize $s_{0},\;{\tilde{u}}_{j}^{*}=0,\;j\leq 0$. 11  **for** j=0:1:N−1
**do**12   Update $s_{j}$ and $x_{j}={\left[z_{j}^{T},{\left({\tilde{u}}_{j-1}^{*}\right)}^{T}\right]}^{T}.$
13   Calculate ${\tilde{u}}_{j}^{*}$ by using ${\tilde{u}}_{j}^{*}=-{\tilde{L}}_{j}x_{j}.$
14  **end**

### 4.3. Angle Deviation Analysis

UAV formation tracking control usually consists of an outer-loop control, including position and velocity control, and an inner-loop control, including pitch/yaw/roll angle control. Many works simply focus on the outer-loop control strategy design. However, the angle deviation is of great importance to UAVs’ motion.

Below, taking an electric quad rotor aircraft as an example, the angle deviation is investigated. In an electric quad rotor aircraft, each motor is attached to a rigid cross frame. Vertical motion and forward/backward motion are controlled by the collective throttle input, i.e., the sum of the thrusts of each motor, and controlling the differential speed of the front and rear motors. The left/right motion of the vehicle is achieved by controlling the differential speed of the right and left motors. The quad-rotor dynamics, evolving in 3D and subject to one force and 3 moments, are modeled by the following equations [13,36].

(35) $$\begin{array}{l} c=f_{1}+f_{2}+f_{3}+f_{4},\\ f_{i}=k_{i}w_{i}^{2},i=1...4,\\ m{\ddot{l}}_{x}=-c\sin\theta ,\\ m{\ddot{l}}_{y}=c\cos\theta \sin\phi ,\\ m{\ddot{l}}_{z}=c\cos\theta \cos\phi -mg,\\ \ddot{\psi }=\zeta_{\psi },\\ \ddot{\theta }=\zeta_{\theta },\\ \ddot{\phi }=\zeta_{\phi }, \end{array}$$

where c is the total thrust produced by four rotors, $f_{i}$ is the force generated by rotor i, $k_{i}\geq 0$ is a constant, $w_{i}$ is the angular speed of motor i, m is the quadcopter’s mass, and g is the gravitational constant. ψ, θ and ϕ represent yaw, pitch, and roll angles, respectively, and $\zeta_{\psi },\;\zeta_{\theta }$, and $\zeta_{\phi }$ are the control inputs for yawing, pitching, and rolling moments, respectively.

Define the state vector as

(36) $$s={\left[l_{x},p_{x},l_{y},p_{y},l_{z},p_{z},\psi ,\dot{\psi },\theta ,\dot{\theta },\phi ,\dot{\phi }\right]}^{T}.$$

The control vector is defined as

(37) $$u={\left[c_{1},c_{2},c_{3},c_{4}\right]}^{T}=\left[c-mg,\zeta_{\psi },\zeta_{\theta },\zeta_{\phi }\right].$$

Then, the dynamics equation can be derived as

(38) $$\left[\begin{array}{c} {\dot{l}}_{x}\\ {\dot{p}}_{x}\\ {\dot{l}}_{y}\\ {\dot{p}}_{y}\\ {\dot{l}}_{z}\\ {\dot{p}}_{z}\\ \dot{\psi }\\ \dot{\psi }\\ \dot{\theta }\\ \dot{\theta }\\ \dot{\phi }\\ \dot{\phi } \end{array}\right]=\left[\begin{array}{c} p_{x}\\ -c_{1}\sin\theta /m-g\sin\theta \\ p_{y}\\ c_{1}\cos\theta \sin\phi /m+g\cos\theta \sin\phi \\ p_{z}\\ c_{1}\cos\theta \cos\phi /m+g\cos\theta \cos\phi -g\\ \dot{\psi }\\ c_{2}\\ \dot{\theta }\\ c_{3}\\ \dot{\phi }\\ c_{4} \end{array}\right].$$

Based on [13,36], the above dynamics equation can be linearized by a Taylor series as

(39) $$\dot{s}\left(t\right)=As\left(t\right)+Bu\left(t\right),$$

where A and B respectively represent the state matrix and input matrix that

$$\begin{array}{l} A=\left[\begin{array}{cccccccccccc} 0 & 1 & 0 & 0 & 0 & 0 & 0 & 0 & 0 & 0 & 0 & 0\\ 0 & 0 & 0 & 0 & 0 & 0 & 0 & 0 & -g & 0 & 0 & 0\\ 0 & 0 & 0 & 1 & 0 & 0 & 0 & 0 & 0 & 0 & 0 & 0\\ 0 & 0 & 0 & 0 & 0 & 0 & 0 & 0 & 0 & 0 & g & 0\\ 0 & 0 & 0 & 0 & 0 & 1 & 0 & 0 & 0 & 0 & 0 & 0\\ 0 & 0 & 0 & 0 & 0 & 0 & 0 & 0 & 0 & 0 & 0 & 0\\ 0 & 0 & 0 & 0 & 0 & 0 & 0 & 1 & 0 & 0 & 0 & 0\\ 0 & 0 & 0 & 0 & 0 & 0 & 0 & 0 & 0 & 0 & 0 & 0\\ 0 & 0 & 0 & 0 & 0 & 0 & 0 & 0 & 0 & 1 & 0 & 0\\ 0 & 0 & 0 & 0 & 0 & 0 & 0 & 0 & 0 & 0 & 0 & 0\\ 0 & 0 & 0 & 0 & 0 & 0 & 0 & 0 & 0 & 0 & 0 & 1\\ 0 & 0 & 0 & 0 & 0 & 0 & 0 & 0 & 0 & 0 & 0 & 0 \end{array}\right],\\ B=\left[\begin{array}{cccccccccccc} 0 & 0 & 0 & 0 & 0 & \frac{1}{m} & 0 & 0 & 0 & 0 & 0 & 0\\ 0 & 0 & 0 & 0 & 0 & 0 & 0 & 1 & 0 & 0 & 0 & 0\\ 0 & 0 & 0 & 0 & 0 & 0 & 0 & 0 & 0 & 1 & 0 & 0\\ 0 & 0 & 0 & 0 & 0 & 0 & 0 & 0 & 0 & 0 & 0 & 1 \end{array}\right]. \end{array}$$

When the network-induced delay ΔT(t) and angle deviations Δψ, Δθ and Δϕ to the yaw, pitch, and roll angles are considered. Assuming the angle deviations are small, the dynamics equation can be further derived based on (38) and (39) as

(40) $$\dot{s}\left(t\right)=As\left(t\right)+Bu\left(t-\Delta T\left(t\right)\right)+\omega \left(t\right),$$

where the disturbance term is given by

(41) $$\omega \left(t\right)=\left[\begin{array}{c} 0\\ -\Delta \theta g\cos\theta \\ 0\\ \Delta \phi g\cos\theta \cos\phi +\Delta \theta g\sin\theta \sin\phi \\ 0\\ \Delta \phi g\cos\theta \sin\phi +\Delta \theta g\sin\theta \cos\phi \\ 0_{6\times 1} \end{array}\right],$$

and here $0_{i\times 1}$ denotes the zero matrix with i×1 size.

Similarly, the corresponding discrete-time dynamics in the j-th sampling interval [jT, (j+1)T) can be derived as

(42) $$s_{j+1}=Es_{j}+D_{j}^{1}u_{j}+D_{j}^{2}u_{j-1}+{\tilde{\delta }}_{j},$$

where ${\tilde{\delta }}_{j}=-\int_{jT}^{\left(j+1\right)T}e^{A\left[\left(j+1\right)T-t\right]}\omega \left(t\right)dtB$ and other parameters are given as the same as in (10).

It is found that the discrete-time dynamics considering angle deviation can be integrated as the same form of (9). Therefore, the proposed control strategy in (23) for the general dynamic control case can still be used to address the problem of angle deviation.

## 5. Simulations and Discussion

In this section, numerical simulations in accordance with real UAV flight data [8,9,32] are given to demonstrate the effectiveness of the proposed UAV formation tracking control strategy in a leader–follower model. In the near-equilibrium case, the quasi-static velocity and fixed desired distance between leader and follower are set. While in the general dynamic case, the desired velocity $p^{*}$ and distance $l_{r}^{*}$ are dynamic. The simulation parameters of both the near-equilibrium case and the general dynamic case are summarized in the Table 2.

**Figure 5:**
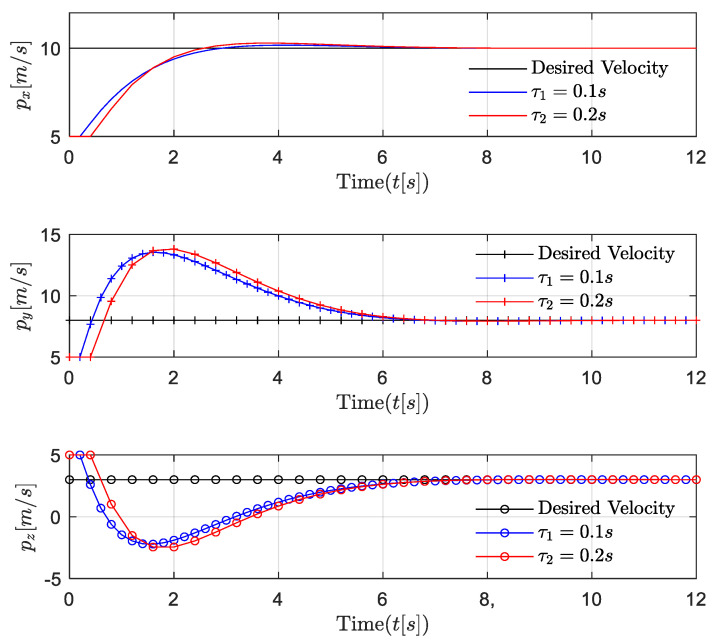
Scenario 1: velocity trajectory comparisons of followers under different delays in near-equilibrium case.

**Figure 6:**
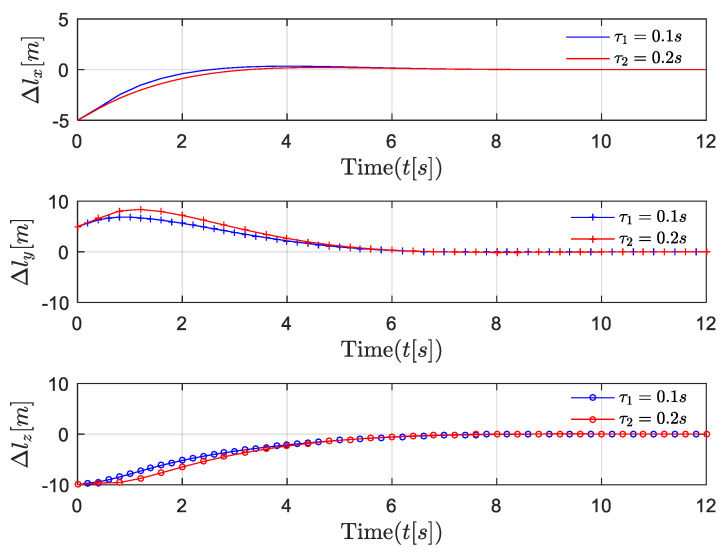
Scenario 1: position trajectory error comparisons of followers under different delays in near-equilibrium case.

**Figure 7:**
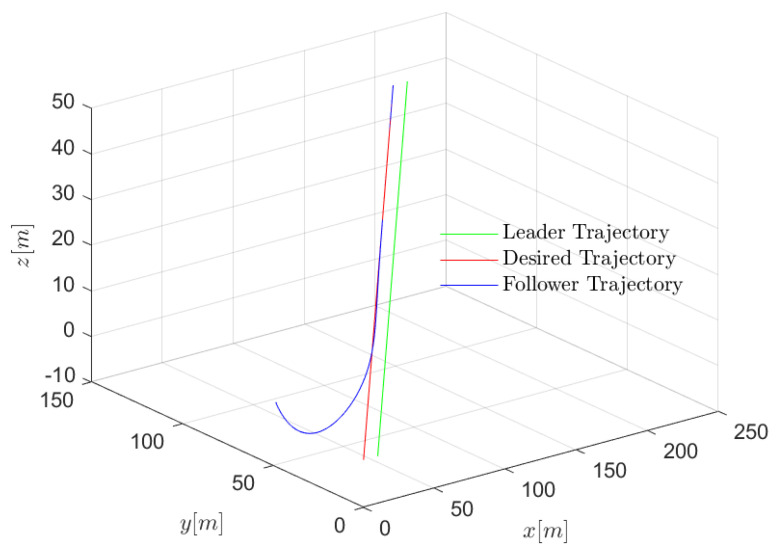
Scenario 1: 3-D trajectory of follower in near-equilibrium case.

First, the position, velocity, and 3D trajectories of followers in the near-equilibrium case are shown in Figure 5, Figure 6 and Figure 7. Afterward, the results are extended to the general dynamic case. In the near-equilibrium case, it can be seen that the velocity and position errors approach zero and that the proposed optimal control algorithm can ensure system stability under various network-induced delays. In addition, we observe that the network-induced delay causes performance degradation of UAV formation tracking because it requires more time for the follower to catch up with the leader when the network-induced delay becomes larger.

To be more specific, in the *x* dimension, the velocity of the follower gradually changes from the initial value of 5 m/s to the desired value of 10 m/s, and the relevant distance error between the leader and the follower reaches 0 step by step as the speed of the follower increases. In the *y* dimension, the velocity of the follower first increases from 5 m/s to the desired value of 8 m/s, while the distance error between the leader and the follower increases to around 8 m because the velocity of the follower is lower than the desired velocity. Then, the velocity of the follower continues to increase to around 13 m/s in order to reduce the distance between the leader and the follower. Finally, the velocity of the follower converges with the desired velocity so that the distance error between the leader and the follower is further reduced to the desired value of 0. Similarly, in the *z* dimension, as the velocity of the follower first decreases and then increases to the desired velocity of 3 m/s, the distance error between the leader and the follower gradually decreases to the desired value of 0. In Figure 6, the same results are shown in the 3D trajectory. The follower can quickly catch up with the leader when there is a sudden velocity and distance error between the leader and the follower, and the follower can maintain the formation’s stability once it successfully follows the leader.

**Figure 8:**
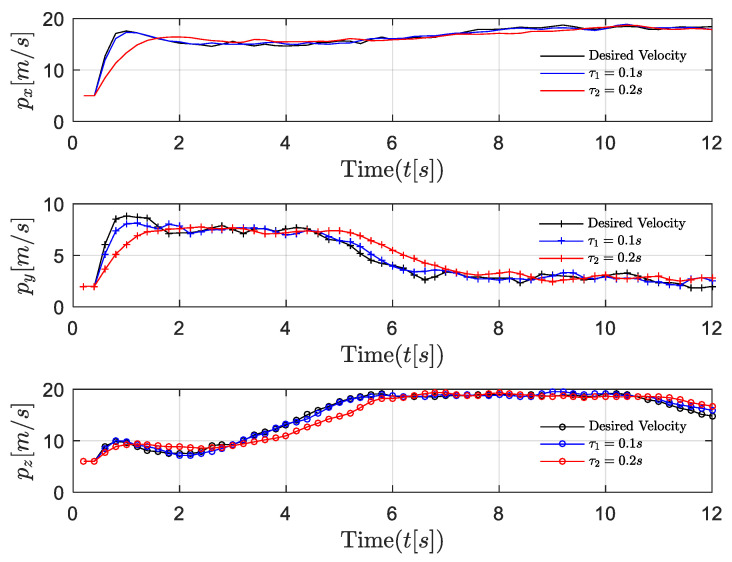
Scenario 2: velocity trajectory comparisons of followers under different delays in general dynamic case.

**Figure 9:**
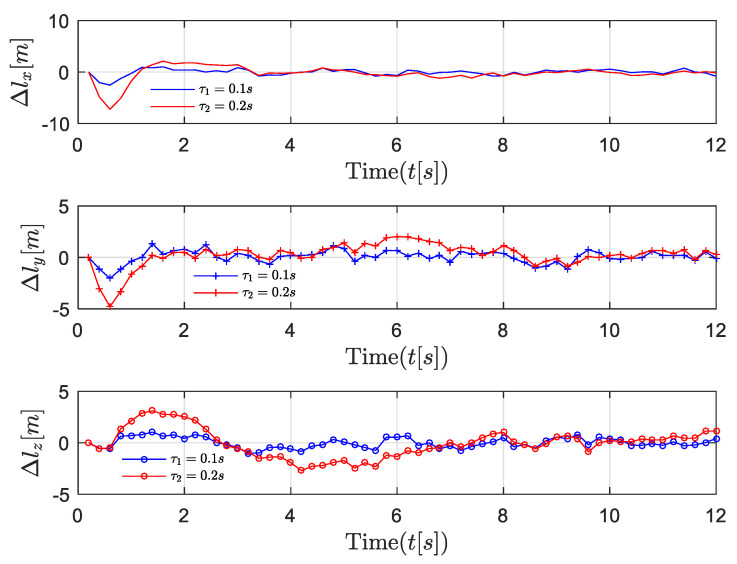
Scenario 2: position trajectory errors comparisons of followers under different delays in general dynamic case.

**Figure 10:**
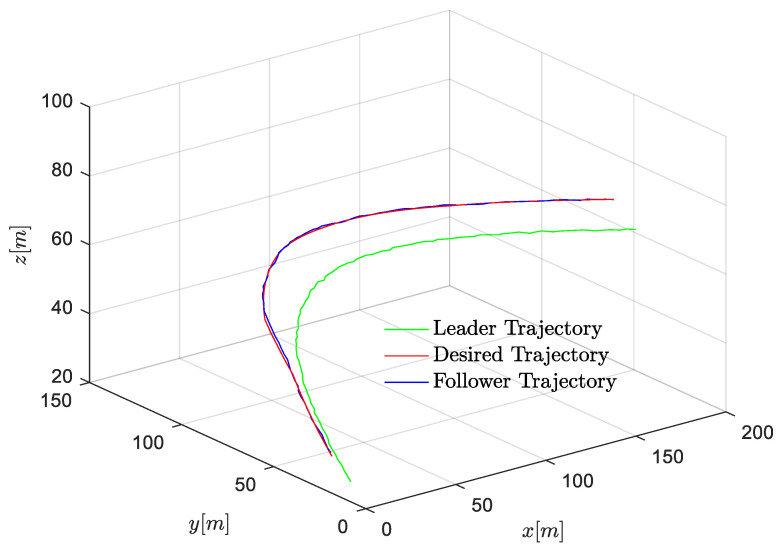
Scenario 2: 3D trajectory of follower in general dynamic case.

The position, velocity, and 3-D trajectories of the follower in the general dynamic case are shown in Figure 8, Figure 9 and Figure 10. It can be seen that the follower can catch up with the leader even if the leader moves with an uncertain time-varying velocity, which indicates that the proposed optimal control algorithm can achieve excellent formation tracking performances in both near-equilibrium and general dynamic cases. However, the dynamic velocity causes serious disturbance to control stability. In the x and y dimensions, at first, the state of the follower remains stable until the velocity of the leader changes. The unstable item $\delta_{j}$ is introduced to the formation system, and the network-induced delay is time-varying as well. Fortunately, the follower still can catch up with the leader in the presence of both the dynamic leader velocity and network-induced delays. Similarly, the performance degradation is introduced by network-induced delay (i.e., the smaller the network-induced delay, the faster the follower reacts).

**Figure 11:**
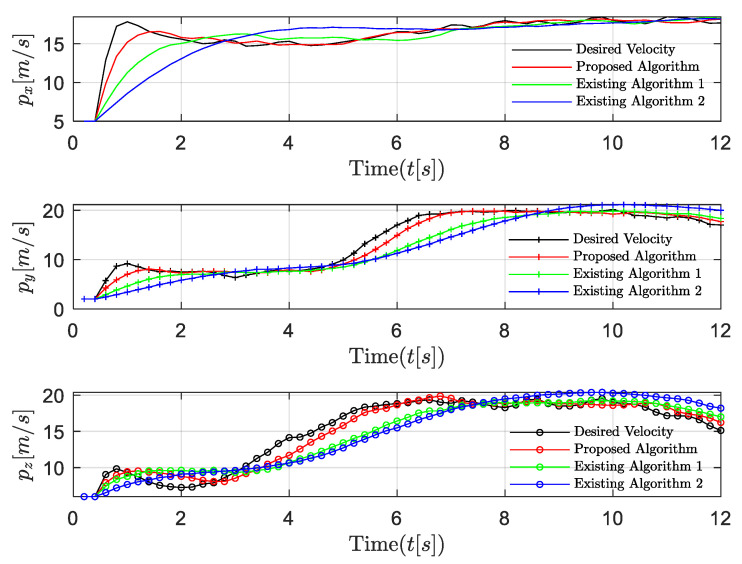
Scenario 2: velocity trajectory comparisons with the existing algorithms.

**Figure 12:**
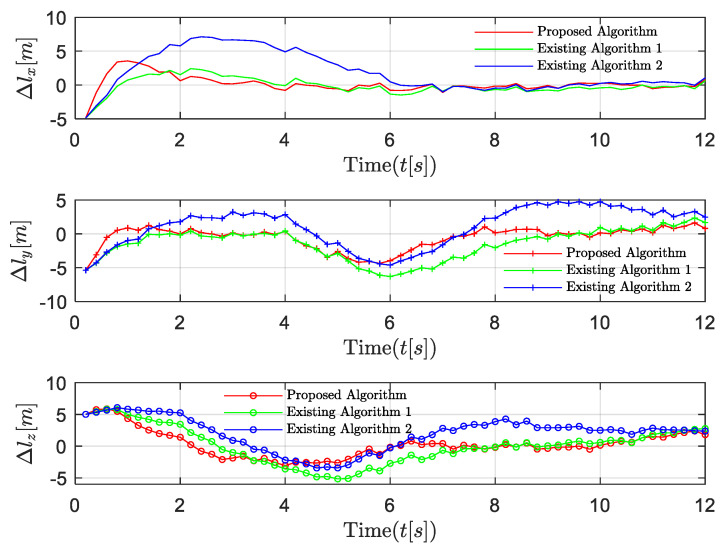
Scenario 2: Position trajectory error comparisons with the existing algorithms.

The performance comparisons between the proposed algorithm and the existing algorithm 1 [34] that ignores network-induced delays and the existing algorithm 2 in [21] that ignores the dynamic feature of the UAV leader are shown in Figure 11 and Figure 12. Compared with the existing algorithms, our proposed algorithm reacts more quickly when the velocity of the leader is changing. This is due to our inclusion of the last control strategy and the uncertain time-varying term in the current control strategy, thus effectively compensating for the influence of dynamic leader velocity and network-induced delays. The algorithm in [21] did not include the time-varying term in the system model and ignored the dynamic features of the system. The algorithm in [34] addressed this problem using a novel approach, but the effect of network-induced delays is still not accounted for. It is obvious that our proposed algorithm provides better stability and faster convergence.

## 6. Conclusions

In this paper, considering the highly dynamic features, including the uncertain time-varying acceleration of the leader and network-induced delays, the optimal control strategy for UAV formation tracking systems is studied. In order to control the follower to achieve the desired state, a linear quadratic optimization problem is proposed with the objective of minimizing the errors between the actual and desired states of the followers. Then, a two-step backward recursion algorithm is developed to address this challenge in a near-equilibrium case, and the results are extended to a general dynamic case. Lastly, the angle deviation is analyzed, and the proposed control strategy algorithm can also be applied to solve such motion problems. Simulated experiments demonstrate the effectiveness of the proposed algorithm. It can be concluded that even if the time-varying acceleration of the leader is uncertain, the follower can still quickly reach the desired state. In addition, the network-induced delay will result in slower reactions by the follower.

## Figures and Tables

**Table 1:** Comparison with existing works.

Ref.	Dynamic Leader Velocity	Delay	Approach	Result
[21]	No	Yes	Neighbor-based linear protocol with time-delay.	A sufficient condition is derived and time-delay cannot be arbitrarily large.
[22]	No	Yes	A piecewise constant and neighbor-based feedback control rule.	A necessary condition is presented and continuous communication between neighboring agents is avoided.
[24]	No	Yes	Finite-field leader–follower consensus protocol with time delays and switching topology.	Two criteria for the finite-field leader–follower consensus with time delays and switching topology are presented.
[25]	No	Yes	An adaptive leader–follower consensus control protocol with unknown nonlinearities and state time-delays.	The consensus tracking error will converge to an adjustable neighborhood of the origin.
[30]	No	No	Three flocking algorithms: two for free flocking and one for constrained flocking.	Migration of flocks can be performed using a peer-to-peer network of agents, i.e., “flocks need no leaders.”
[31]	Yes	No	Flocking of multi-agent protocol with a virtual leader.	Modification to the Olfati-Saber algorithm in [30].
[33]	No	Yes	Consensus-based approaches are applied to achieve time-varying formation.	Necessary and sufficient conditions for UAV swarm systems to achieve time-varying formations are proposed.
[34]	Yes	No	A continuous adaptive controller is designed.	An adaptive estimator for each uninformed agent can estimate the velocity of the leader.
[35]	Yes	Yes	An adaptive leader–follower formation control protocol is proposed.	The overall closed-loop system is proved to be semi-globally, uniformly, and ultimately bounded by Lyapunov stability theory.

**Table 2:** Simulation parameters setting.

Parameter	Scenario 1 Near-Equilibrium Case	Scenario 2 General Dynamic Case
Sampling period	0.4 s	0.4 s
Network-induced delays	τ∈[0.1 s,0.2 s]	τ∈[0.1 s,0.2 s]
Desired velocity	Fixed $\left(p_{x},p_{y},p_{z})=(10,8,3\right)$ m/s	Dynamic, average velocity: 15 m/s
Desired distance	Depend on velocity	Depend on velocity
Uncertainty	None	Disturbance distribution N(0,1)

## Data Availability

The data presented in this study are available on request from the corresponding author.

## Appendix A

Below, the derivation from (6) to (9) is shown. First, based on (6), multiplying both sides of the equation by an exponential term as

(A1) $$e^{-At}\dot{s}\left(t\right)=e^{-At}As\left(t\right)+e^{-At}B\left[u\left(t-\Delta T\left(t\right)\right)-g_{r}\left(l_{r}\left(t\right),p_{r}\left(t\right)\right)\right].$$

Then, we have

(A2) $$\frac{de^{-At}s\left(t\right)}{dt}=e^{-At}B\left[u\left(t-\Delta T\left(t\right)\right)-g_{r}\left(l_{r}\left(t\right),p_{r}\left(t\right)\right)\right].$$

Integrating both sides of (A2) as

(A3) $$\int_{jT}^{(j+1)T}\frac{de^{-At}s\left(t\right)}{dt}=\int_{jT}^{(j+1)T}e^{-At}B\left[u\left(t-\Delta T\left(t\right)\right)-g_{r}\left(l_{r}\left(t\right),p_{r}\left(t\right)\right)\right].$$

Then, we have

(A4) $$s_{j+1}=e^{AT}s_{j}+\int_{jT}^{(j+1)T}e^{A[(j+1)T-t]}B\left[u\left(t-\Delta T\left(t\right)\right)-g_{r}\left(l_{r}\left(t\right),p_{r}\left(t\right)\right)\right]dt.$$

In a sampling interval, the control strategy is piecewise and includes two parts due to network-induced delays. During $\left[jT,jT+\Delta T_{j}\right)$ and $\left(jT+\Delta T_{j},\;(j+1)T\right]$, the control strategy is $u_{j-1}$ and $u_{j}$, respectively. Then, the formula (A4) can be written as

(A5) $$s_{j+1}=e^{AT}s_{j}+\int_{jT}^{jT+\Delta T_{j}}e^{A[(j+1)T-t]}Bdtu_{j-1}+\int_{jT+\Delta T_{j}}^{(j+1)T}e^{A[(j+1)T-t]}Bdtu_{j}-\int_{jT}^{(j+1)T}e^{A[(j+1)T-t]}g_{r}\left(l_{r}\left(t\right),p_{r}\left(t\right)\right)dtB.$$

The discrete-time dynamics can be further obtained as

(A6) $$s_{j+1}=e^{AT}s_{j}+\int_{0}^{T-\Delta T_{j}}e^{At}dtBu_{j}+\int_{T-\Delta T_{j}}^{T}e^{At}dtBu_{j-1}-\int_{jT}^{(j+1)T}e^{A[(j+1)T-t]}g_{r}\left(l_{r}\left(t\right),p_{r}\left(t\right)\right)dtB,$$

which is equivalent to the discrete-time dynamics in (9).
